# Development and feasibility testing of an intervention to support active lifestyles in youths with type 1 diabetes—the ActivPals programme: a study protocol

**DOI:** 10.1186/s40814-016-0106-7

**Published:** 2016-11-08

**Authors:** Fiona Mitchell, Alison Kirk, Kenneth Robertson, John J. Reilly

**Affiliations:** 1 Physical Activity and Health Group, University of Strathclyde, Glasgow, Scotland; 2 Children’s Diabetes Service, Greater Glasgow and Clyde, Yorkhill Hospital, Glasgow, Scotland

**Keywords:** Type 1 diabetes, Physical activity, Intervention, Youths, ActivPals

## Abstract

**Background:**

The global incidence of type 1 diabetes is rising, and youths with type 1 diabetes continue to suffer poorer health than peers without diabetes. Evidence suggests youths with type 1 diabetes have physical activity (PA) levels well below the recommendations for health and have high levels of sedentary behaviour. An active lifestyle is therefore recommended to improve health. There is limited research showing effective lifestyle behaviour change in this population; therefore, an evidence gap exists between the need to promote physical activity in type 1 diabetes care and lack of understanding on how to do this. This protocol paper describes a feasibility and pilot study of the ActivPals programme—an intervention to support active lifestyles in youths with type 1 diabetes.

**Methods/design:**

Key intervention components have been identified from preliminary work (individual and family focus, peer mentoring, technology integration and improved communication and understanding) and are being developed into a pragmatic randomised controlled trial (RCT) supported by recruitment pathways. A steering group of health care professionals and managers will refine the intervention to patient needs. A pilot trial is providing data on intervention implementation, acceptability and feasibility. Twenty youths with type 1 diabetes are being recruited and randomised into an intervention or control group. Physical activity is being measured objectively using the Actigraph GT3X+ monitor at baseline and 1-month follow-up. Contextual factors associated with intervention delivery are being explored.

**Discussion:**

This study will contribute to the development of evidence-based, user-informed and pragmatic interventions leading to healthier lifestyles in youths with type 1 diabetes.

## Background

Type 1 diabetes is a chronic disease where the insulin-producing pancreatic beta cells are destroyed resulting in an inability of the body to regulate blood glucose. The condition is managed by regular monitoring of blood glucose, administering insulin and participating in a healthy diet and regular physical activity [1]. Diabetes has been a growing public health burden across the world [2] with treatment for type 1 diabetes costing the NHS in England roughly £1.802 billion a year [3]. The global incidence of type 1 diabetes is rising with an estimated 70 % rise in the disease by 2020, in European adolescents under age 15 [4]. Despite significant improvements in technology for blood glucose management, youths with type 1 diabetes continue to suffer from poorer health, relative to peers without diabetes. For example, research suggests there are higher mortality rates, more cardiovascular risk factors, higher rates of depression, lower educational attainment and poorer psychosocial health outcomes [5, 6].

Glycosylated haemoglobin (HbA1c) is a measure of glycaemic control and is considered by both patients and health care professionals to be at the core of type 1 diabetes management [7]. HbA1c is an important marker for risk of developing micro- or macrovascular complications of diabetes (such as retinopathy, nephropathy, cardiovascular disease and cerebrovascular disease) [8]. Diabetic complications develop as a result of chronic hyperglycaemia which causes damage to tissues and can develop as early as 2 years from diagnosis [9]. Improving HbA1c is therefore a priority for youths with type 1 diabetes.

There is now a small body of evidence which suggests regular physical activity (PA) can significantly reduce HbA1c levels in individuals with type 1 diabetes [7, 10, 11]. This builds on the prolific evidence showing the positive physical and psychological benefits of regular PA in childhood and adolescence [12]. Regular physical activity is therefore recommended in clinical guidelines as one of the core elements of good type 1 diabetes management [13]. Despite appreciation for the benefits of physical activity, evidence suggests youths with type 1 diabetes are less physically active than peers without diabetes [14–16]. For example, a recent study [16] found that young people with type 1 diabetes aged 7–9 and 12–14 years spent on average 78 % (10.2 h/day) of the waking day sedentary and 43 min/day participating in moderate to vigorous PA. Sedentary behaviour is a distinct class of behaviours (i.e. sitting, watching television, playing video games) that is characterised by little physical movement and low energy expenditure [17]. Only two of the 40 participants in the study achieved minimum guidelines of PA participation of 60 min moderate to vigorous intensity PA (MVPA) on each accelerometer wear day, and 19/40 did not achieve 60 min of MVPA on any day. As PA levels are well below the recommendations for health and sedentary behaviour is high in youths with type 1 diabetes, there is clearly a need for intervention studies to support this population to lead an active lifestyle.

Whilst there have been a variety of published intervention studies with youths with type 1 diabetes, there are limitations with this work. For example, interventions have not been based on behavioural change theories [10, 18], have uncontrolled designs [19, 20], or consist of a very structured supervised intervention design (e.g. using supervised structured exercise classes in the intervention) [20–24]. Whilst short-term changes in PA might be evident with such supervised settings, often, the PA behaviour reverts back to pre-intervention levels when the supervised intervention is removed [25]. No study has been conducted, to our knowledge, which is theoretically based, adopts a randomised controlled design and has been tailored to young people and families with type 1 diabetes to support long-term lifestyle behaviour change. As such, there is a need for new, higher quality (evidence informed and theoretically based) interventions, which are developed using the UK Medical Research Council (MRC) framework [26].

**Fig. 1:**
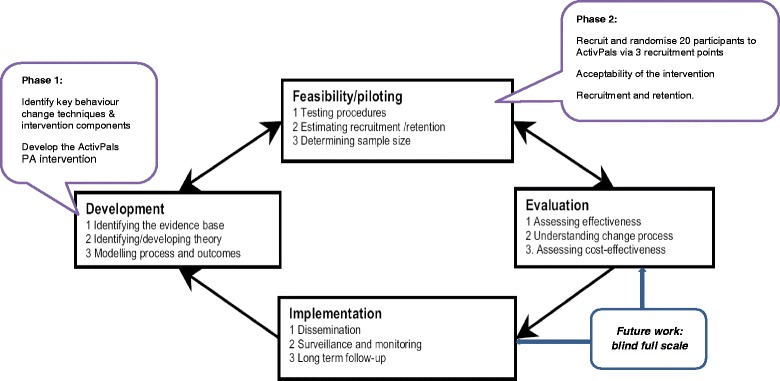
MRC framework for designing and evaluating complex interventions

The MRC framework for evaluating complex interventions will be used as the basis for this research [26]. The framework strongly advises carrying out feasibility and pilot work prior to running a full-scale trial; therefore, in keeping with phases 1 and 2 of the MRC framework, this study proposes to examine the feasibility of recruitment, retention and acceptability of an RCT PA intervention for youths with type 1 diabetes (see Fig. 1). This study aims to (1) develop a theoretically based tailored lifestyle intervention (ActivPals programme) to support active lifestyles for youths with type 1 diabetes and to (2) explore the feasibility of delivering this intervention. The ActivPals programme is being developed from previous work with youths with type 1 diabetes, which highlighted the importance of peer and parental support when leading an active lifestyle. This work will provide critical information for the development of a definitive trial, in addition to providing important information for improving clinical care of type 1 diabetes in youths. The intervention consists of two key phases:

Phase 1 specific objectives are to develop:

1. An intervention (ActivPals programme) to support active lifestyles in youths with type 1 diabetes
2. Feasible recruitment pathways towards the intervention

Importantly, the intervention and recruitment pathways will be evidence based whilst being pragmatic and suitable for integration within current type 1 diabetes NHS practice.

Phase 2 specific objectives are to recruit participants and conduct a pilot and feasibility trial to determine:

1. The recruitment, initial retention and adherence level that can be achieved for a 4-week intervention programme in both the intervention and control groups.
2. Preliminary evidence of effects of the intervention on physical activity, sedentary behaviour and quality of life. This will provide a preliminary indication of whether the intervention can show change within this group. Following this, an effect size will be estimated for a ‘definitive’ randomised controlled trial.
3. The acceptability of the intervention recruitment pathways and intervention content, delivery, duration and intensity to participants and health professionals.

## Methods

### Study population

Participants will be included in phase 1 of the research if they meet the following inclusion criteria:

1. Aged between 7 and 16 who have a medical diagnosis of type 1 diabetes (a medical professional will have previously tested glycated haemoglobin (HbA1c) levels and provided a diagnosis)
2. Are registered in Greater Glasgow and Clyde Children’s Diabetes Service
3. Are independently ambulatory

Parents and carers of possible participants will also be invited to support the individual in participation. Individuals will not be eligible to participate in the study if they have:

1. Been advised not to undertake physical activity by their doctor
2. Severe learning disabilities and not able to understand the study protocol
3. Severe challenging behaviour or other needs requiring constant one to one support

### Recruitment

Researchers have identified the need for a recruitment strategy in RCTs [27, 28]. A strategy has been designed to guide the recruitment process. The full strategy is shown in Appendix. Participants will be recruited to phase 2 via three recruitment points: (1) from paediatric diabetes clinics (main recruitment site), (2) through support groups or clubs for young people with type 1 diabetes and (3) the diabetes nurses working at the hospital will screen the medical records of paediatric patients registered at the clinic for eligibility to participate in the study. To ensure patient confidentiality, clinic staff will screen the patient records and only retrieve information on participant’s age, any exclusion criteria and next appointment date. The staff and researcher will not discuss any information on patient files. The researchers will assess the most effective recruitment routes which will inform future work with this population. There are three type 1 diabetes clinics per week at Yorkhill hospital (the main recruitment site) and others across Greater Glasgow and Clyde paediatric diabetes service. Greater Glasgow and Clyde is the largest urban area in Scotland and the fifth largest in the United Kingdom (UK) [29], therefore offering a fairly representative sample of young people living in urban areas in the UK. Participants who attend a paediatric diabetes clinic and meet the inclusion criteria will be informed of the study by the researcher or by the paediatric consultant, diabetes doctors and nurses. Those who are eligible and interested in participating in the study will be given an information pack which will include more details about participation. Participants can express interest in the study by signing and returning a tear-off slip in the information pack and posting it using the self-addressed envelope provided. The researcher will then contact participants and arrange a visit to discuss the study. A strong collaboration between the research group and the diabetes health care team has already been established based on previous research carried out with this population [10, 16].

### Consent and randomisation

Participants who are interested and eligible to participate will be randomised individually to the intervention or control group. As this is a small-scale feasibility and pilot study, the researcher collecting the data will also deliver the intervention. Therefore, it is not possible for the researcher to be blind from the treatment group. Another member of the research team (the PI) will randomise participants and write the treatment allocation for each participant on a piece of paper. This will be placed in a sealed envelope, only to be opened by the researcher immediately before the intervention/control group visit. Consent/assent will be sought at two stages: firstly, for permission from the carer to be contacted by the researcher to arrange appointments and, secondly, written consent/assent to opt into participate in each aspect of the study (physical activity intervention component and interview component). This will be sought at the study visits, once the researcher has discussed the study in detail with participants. Written information sheets will be given to participants (young people and parents/carers). The researcher will go over the information sheet with participants at the first visit to ensure that participants understand the study protocol and what is being asked of them.

### Withdrawal of study participants

The participants will be given every opportunity to clarify points they do not understand and, if necessary, ask for more information. Participants will be given sufficient time to consider the information sheets provided. It will be emphasised that the participant may withdraw their consent to participate at any time without loss of benefits to which they otherwise would be entitled. Participants will only be withdrawn from the study by the researcher if the researcher perceives them to be at risk or if there is a serious adverse event. If there is a serious adverse event (e.g. injury from exercise, medical help sought for diabetes), the details of this will be recorded on a Serious Adverse Event form, provided by the local NHS board and national good clinical practice will be followed. The researchers will monitor any adverse events during the study.

## ActivPals intervention

The ActivPals intervention aims to:

- Support youths with type 1 diabetes to initiate and maintain an active lifestyle, including increased MVPA and reduced sedentary behaviour
- Be endorsed by NHS diabetes care staff highlighting a clear integration of physical activity into diabetes care and be of a duration and intensity realistic for roll out in practice

**Fig. 2:**
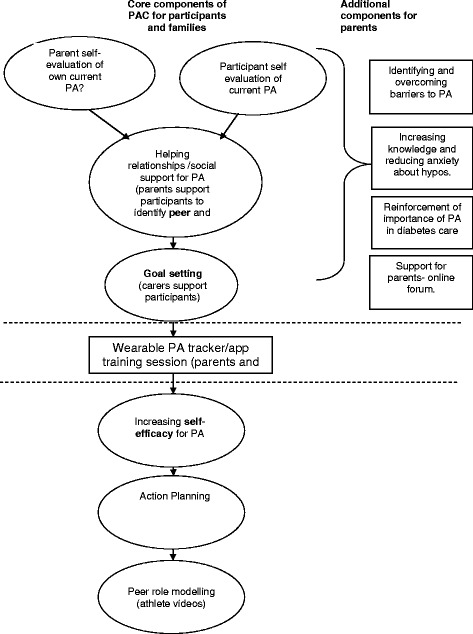
ActivPals PA intervention components

The theoretical framework for the intervention will draw on Social Cognitive Theory [30], which emphasises the importance of self-efficacy and setting realistic goals. Models of peer support [31], defined as ‘support from a person who has experiential knowledge of a specific behaviour or stressor and similar characteristics as the target populations’, will also be the key to the intervention. The ActivPals intervention will be tailored to the individual’s baseline activity, activity preferences and local opportunities. The inclusion of one or two parents or carers or other support person throughout the full intervention period will be strongly encouraged. The intervention will be delivered by the researcher (first author) who is collecting the data for the study. The intervention consists of (1) an initial physical activity consultation [32] incorporating behaviour change techniques and (2) the use of role modelling/peer mentors (athletes and roles models with type 1 diabetes have endorsed the research and have provided video messages to support young people with type 1 diabetes to be more active). The third key component of the intervention is the use of a wearable self-monitoring device which syncs to a mobile app and website. Continued support using social media/electronic SMS text messages, telephone contact or email will also be made by the researcher to encourage participants to adhere to their physical activity plan. See Fig. 2 for a diagram of the intervention components.

### Phase 1 (4 months): aim—development of intervention and recruitment pathways

The researchers have identified the key components of the intervention that will be developed and piloted based on prior work with this population. A steering group of diabetes health care professionals and individuals at management level will be established, using the James Lind Alliance framework. The steering group will advise on how these key components are implemented and will assist with refining the intervention. The steering group will help to guide the intervention to the needs of patients, tailor intervention delivery within current clinical practice and support dissemination to a broader audience of patients and diabetes educators.

### Physical activity consultation

The researchers have carried out previous work to identify important components of an intervention [10, 16]. This includes incorporating behaviour change techniques, education and support for diabetes preparation and a combination of group and one to one support. A physical activity consultation aligns well with these identified components and has been successfully used with other diabetes groups [32]. Guidelines on conducting this consultation have been published for use in adults with diabetes [32]. The consultation will involve an individual or group discussion around physical activity and sedentary behaviour and aims to increase motivation, provide education and develop an individual tailored plan for supporting an active lifestyle. Strategies and techniques identified as important for supporting behaviour change are incorporated to support initiation and maintenance of an active lifestyle. Examples of strategies which may be included are investigating current physical activity behaviour; discussing benefits, barriers and costs of becoming more active; identifying suitable activities; establishing and enhancing social support and self-efficacy; setting personal goals and discussing relapse prevention (Fig. 2).

**Fig. 3:**
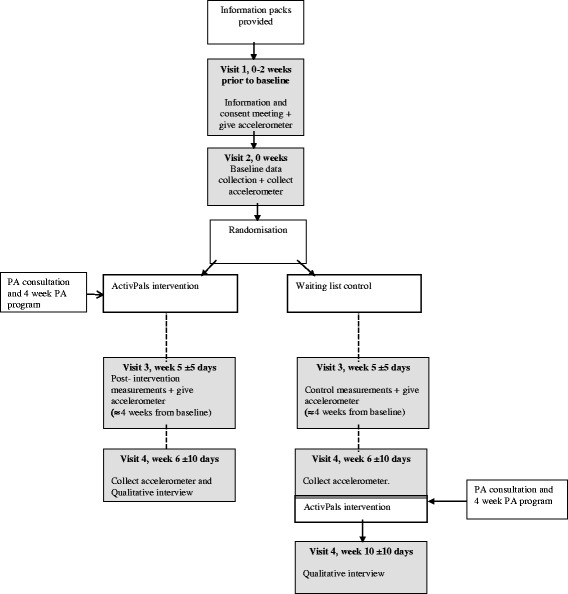
Study flow chart

### Role modelling/peer mentors

Often, group structured education sessions are offered as part of diabetes care. Group/peer support has been identified as an important component of an intervention to support active lifestyles in our previous research [10, 16]. During the development of the ActivPals intervention, we will explore how the intervention could be integrated with current diabetes group education sessions. In particular, group sessions offer an opportunity to incorporate peer support, involving sharing experiences, mentoring and role-modelling. The suitability and feasibility of including peer support within group educations will be explored during intervention development. In addition, the inclusion of role models/peer mentors will be examined. Peer mentors will be athletes with type 1diabetes identified by the researchers. Motivational videos will be provided as part of the intervention, which will contain information about the benefits of physical activity participation, particularly when living with type 1 diabetes. Participants will be given the link to the ‘YouTube’ videos and encouraged to watch these regularly in their own time, over the intervention period, to increase PA.

### Continued support through social media/emails or telephone contact

We will explore the possible use of social media, emailing or telephone support as a mechanism for continued support and to provide further information on aspects of diabetes management.

### Phase 2 (8 months): aim—feasibility trial

Once the ActivPals intervention has been developed, we will conduct a feasibility study to explore intervention feasibility and test practical aspects of study design. Based on previous literature, an effect size will be estimated for a subsequent definitive randomised controlled trial.

### Pilot procedures

Participants (child and parent/carer) will be visited a minimum of four times during this pilot study. During visit 1, basic demographic (gender, age), medical information (body mass index, diabetes duration and current therapy), PA and QoL questionnaires (see primary and secondary outcome measures section) will be completed and participants will be given an Actigraph activity monitor and asked to wear this for the next 7 days. An accelerometer wear diary will be given to participants to record attachment and removal.

Visit 2 will occur approximately 8 days later. At this point, activity monitors and wear diaries will be returned and participants will then be randomised into the intervention or control group. At this point, the researcher will open the envelope to reveal the treatment group. Those who have been allocated to the intervention arm of the study will receive the PA consultation and intervention materials. The intervention period will last 4 weeks, after which visit 3 will take place. During this visit, both intervention and control participants will once again be given an Actigraph activity monitor and asked to wear this for the next 7 days. An accelerometer wear diary will again be given to participants to record attachment and removal. Visit 4 will occur approximately 8 days later during which time activity monitors and wear diaries will be returned. Participants will be asked to complete the PA and QoL questionnaires with the researcher. Intervention participants will be invited to participate in a short interview to explore views on the ActivPals intervention programme. At this point, participants in the control group will be offered the physical activity intervention, followed by an interview. A wait list control design will be used as it would be unethical to deny participants’ access to an intervention which we believe is likely to be beneficial to health. See Fig. 3 for study flow chart. More details on the pilot study design are noted below following the PICOT protocol (e.g. Population, Intervention, Comparison group, Outcome measures (feasibility and patient centred) and Timing).

#### Population

See the ‘Study population’ section.

#### Intervention

The ActivPals intervention programme will be developed during phase 1.

#### Comparison group

The comparison group will receive standard diabetes care. Control participants will be offered the intervention after they have completed follow-up outcome measures.

#### Feasibility outcome measures

We will monitor the feasibility of the trial by tracking recruitment, retention and adherence rates of participants and the most effective points of recruitment. In addition, feedback about the delivery of the intervention, intervention content and perceived impact will all be explored through the qualitative interviews with participants and parents, carried out at the end of the study. The researchers will also meet with the steering group at the end of the study to feedback the results and discuss intervention acceptability.

#### Patient-centred outcome measures

Objective measures of physical activity and sedentary behaviour will be collected using the Actigraph GT3X+ monitor. This monitor will allow objective recording of daily time spent in sedentary, light and moderate to vigorous physical activity. These monitors are small (approx size of a 2-pound coin) and lightweight (19 g). Participants will be asked to wear the accelerometers around the waist during waking hours for 7 days, excluding water-based activities. Accelerometer data will be downloaded to Actilife software (version 6.4.3). In line with previous studies, a minimum wear time for a valid day will be defined as 6 h/day, with 3 days of data required for analysis inclusion [33, 34].

The primary outcome measure of daily time spent in MVPA and sedentary behaviour will be determined using cut-points calibrated and validated in paediatric studies: sedentary (<100 cpm) [35] and MVPA (≥3200 cpm) [36]. In addition to the accelerometer data, information will be gathered about the type, frequency and location (e.g. school) of activities undertaken in the last 7 days. This information will be collected from participants with the help of their parent/cares at baseline and follow-up (post-intervention/control). This questionnaire will be developed based on the findings of a previously conducted survey study [37].

Generic and disease-specific questionnaires will be used to measure quality of life in participants. The PedsQoL 4.0 Generic Core Scale was used to measure general quality of life [38]. This 23-item questionnaire contains the following subscales: physical functioning, emotional functioning, social functioning and school functioning. A psychosocial health summary score will be calculated from the average of the emotional, social and school functioning subscales, a physical health summary score (from the physical functioning subscale) and a total overall score from the average of all subscales. This scale has shown good reliability and validity in this population [38, 39]. The PedsQoL 3.0 type 1 Diabetes Module is a 28-item questionnaire measuring diabetes-specific QoL consists of five subscales: diabetes symptoms, treatment barriers, treatment adherence, worry and communication. Patients (self-report) and their parents (proxy-report of the child’s QoL) will complete questionnaires by rating items on how much each was a problem in the previous month using a 5-point Likert scale (‘0’ = never a problem; ‘4’ = almost always a problem). This questionnaire has been validated and has shown to be reliable in children with type 1 diabetes [38, 40]. Changes in general QoL and diabetes module scores will be analysed between intervention and control groups to asses for any trends in intervention effects.

We will also determine key process-related outcomes including intervention recruitment, retention and compliance. As mentioned, a qualitative interview will be carried out post-intervention to determine contextual factors associated with delivery of the intervention and to explore patient and health professional experiences of the ActivPals programme including acceptability of procedures, perceived benefits and difficulties. According to the MRC framework [41], qualitative research can be valuable for identifying what the important or ‘active ingredients’ of an intervention are and which elements are not related to the ‘treatment effect’. Topics which will be covered include perceptions of project, sport and PA participation, views on intervention components, attitudes towards PA, benefits and barriers towards PA and sustainability of PA. A parent is encouraged to participate in the interview. With the participant’s permission, the interviews will be recorded and transcribed. Otherwise, notes taken during the interview will be written up in detail as soon as possible afterwards. Interviews will be analysed by thematic analysis. Feasibility and acceptability measures will be reported including programme implementation and fidelity to protocol.

The measures will give an indication of the effects of the intervention on PA levels, sedentary behaviour and quality of life. The acceptability of the measures and missing data will be considered when designing the full-scale trial.

#### Timing

Outcome measures will be assessed at baseline (before intervention) and 1 month after the initial physical activity consultation appointment.

This pilot RCT will be performed according to the Research Governance Framework for Health and Community Care (second edition, 2006).

### Statistics and data analysis

Descriptive statistics will be presented (mean and standard deviation) with 95 % confidence intervals presented for each group separately. Changes in physical activity and QoL from baseline and follow-up will be assessed using an ANOVA. As this is a feasibility and pilot study, the study will be underpowered; therefore, quantitative outcomes will be interpreted only as feasibility and pilot data.

## Discussion

This paper describes the ActivPals study aims and design, including information about the intervention, the outcome measures and recruitment process. Whilst there is a strong evidence base which suggests that regular PA can have a range of physical and psychological benefits for youths with type 1 diabetes, an evidence gap exists between the need to promote long-term lifestyle physical activity in type 1 diabetes care and lack of understanding on how to do this. A vital first step in developing this field is development and piloting a theoretically based, pragmatic, lifestyle intervention for youths with type 1 diabetes.

### Limitations of the research

As this is a small-scale pilot and feasibility study, the researcher (first author) will be recruiting participants, collecting the data and also delivering the intervention. Whilst we acknowledge that a double-blind pilot RCT would strengthen the design of the study, there is limited time and resource with this small-scale pilot study. Therefore, any future funding applications to test the effectiveness of the intervention would include costs for a health professional and research assistant to avoid potential biases in the trial design. Researcher bias will be reduced in the qualitative element of the study as an MSc student, independent to the study, will be conducting the interviews.

The time and funding limitations also restrict the possibility of collecting post-intervention/control follow-up data. Thus, it is not possible to assess long-term effects of the intervention in this small pilot study. These limitations will be addressed in the next phase of work.

### Perceived risk

As the aim of the intervention is to increase physical activity levels, changes to lifestyle are encouraged. There is unlikely to be any pain or discomfort associated with increased physical activity. Participants will be advised to carry out prescribed stretches before and after the activity to minimise any muscle stiffness resulting from exercise. These will be described in detail by the researcher when delivering the intervention. Increased exercise can cause hypoglycaemia in those with type 1 diabetes, if they are experiencing low blood sugar levels. Participants will therefore be asked to monitor blood sugar levels before and during exercising, and post exercise, to minimise the risk of this occurring. Participants will follow Greater Glasgow and Clyde children’s diabetes service exercise guidelines, which will be included in the intervention information books for parents and young people with type 1 diabetes (intervention resources). The researcher will talk participants through this information during the PA consultation. The intervention will be individualised to each participant’s baseline level of activity; therefore, the activity will begin at a level that is comfortable and achievable. Activity intensity, frequency and duration will increase progressively over time. The intervention will be designed by experts working in the physical activity and diabetes field who have experience of advising on appropriate levels and intensity of activity. At the end of the study, the resources will be integrated with current routine care for type 1diabetes (i.e. these will be offered to all patients when they are visiting clinic). Participants will also be given information about support networks and other diabetes care team members they can speak to about increasing physical activity.

### Strengths of the research

A key strength of this study is that it aligns with the development and feasibility stage of the MRC framework for the development of complex health interventions. The findings from this feasibility and pilot study will generate output critical to the subsequent stage of the MRC framework which is the development and running of a definitive trial exploring the effectiveness of physical activity and sedentary behaviour intervention within type 1 diabetes care (see Fig. 1). Specific output from phase 1 will be a new evidence-based, pragmatic and user-informed intervention and suitable recruitment pathways to support active lifestyles for youths with type 1 diabetes. Specific output from phase 2 will be an important information on recruitment, initial retention and the adherence level that can be achieved for a 4-week intervention in both the intervention and control groups. In addition, indicative effect of the intervention on physical activity and sedentary behaviour will be important for the definitive trial. Acceptability of the intervention, recruitment pathways and intervention content, delivery, duration, intensity to participants and health professionals will also be explored. The study will therefore lead to new knowledge of direct relevance to the NHS for improving physical activity both in diabetes care and in the care of youths with other chronic conditions. Importantly, the qualitative interviews will provide information about context of young people with type 1 diabetes lives and will also allow us to understand experiences, attitudes, perceptions and behaviours following completion of the ActivPals PA intervention. This exploratory work will be instrumental in designing and developing a full-scale trial to test the effectiveness of the intervention.

The study is currently under way. All participants were recruited from January to March 2016. Results of the study will be submitted for publication from January 2017.

## Appendix

### Recruitment strategy framework

Stage 1—pool

- Identify target group within population or setting
  Young people aged 7–16 with type 1 diabetes registered at Greater Glasgow & Clyde Paediatric diabetes service.
- Formative evaluation of recruitment approaches
  A multipoint recruitment strategy will be used to recruit from three main sources:
  - GG & C paediatric diabetes clinics (main recruitment site)
  - New start (newly diagnosed) groups or support groups or clubs for young people with type 1 diabetes
  - Screen medical records of paediatric patients registered at the clinic for eligibility to participate in the study

Stage 2—invited

- Offer invitation (January 2016–March 2016)
  Information packs will be given out/sent to the paediatric diabetes service and support groups by the research associate (RA). These will contain a letter introducing the study and information sheets for:
  - Children aged 7–11
  - Young people aged 12–16
  - A relative/parent
  If participants would like to be contacted by the RA with more information about the study, they are invited to sign and return the tear-off slip in the self-addressed envelope provided.
- Monitor response uptake (January 2016–March 2016)
  As monitoring the responses allows the researchers to evaluate the effectiveness of the recruitment strategy (Foster et al., 2011), the RA will monitor how many tear slips are returned and where the participants were recruited from. This will allow the team to asses to most effective recruitment point.
  Telephone reminders have been identified as an effective strategy for recruitment (Treweek et al., 2010); therefore, the researcher will phone participants who have not returned their tear-off slip within a 2-week period. This active method will serve as a reminder to participants and facilitate awareness of the study. Participants can inform the researcher if they want more information about the study or if they do not want to take part in the study. If the information pack has been lost, the researcher will send out another information pack.
  Once all of the participant slips have either returned forms or have confirmed to the research secretary that they do not want to take part in the study, the research team will review the number of consenting participants and assess the success of the recruitment strategy.

Stage 3—responded (January 2016–March 2016)

Participants who requested more information about the study will be contacted by the RA to agree a date and time for a home visit. This date/time will also be agreed with a parent or nominated carer to ensure they also receive information about the study.

- Re-invitation to responders before intervention begins
  Foster et al. (2011) suggest that recruitment and retention to PA studies can be strengthened if participants are invited to participate face to face. Thus, the RA will visit each interested participant in their home (or alternative venue if preferred by participant) to provide more information about the study. Consent forms will also be given to the participants and parent/carer. Consent forms can either be filled in whilst the RA is present or these can be left with participants to allow them time to consider their participation. If the participant is 11 years or younger, a parent/carer must provide consent on the young persons’ behalf, agreeing that they will support the participant to take part in the programme. There will also be an opportunity for participants, parent and carers to ask questions pants about the study.
- Facilitate attendance
  Evidence suggests that greater contact between trial advisors and recruiting sites may increase recruitment (Liénard 2006; Monaghan 2007). Therefore, the researcher will carry out follow-up phone calls to interested participants. These will also act as reminders to participants, parents/carers who have not returned consent forms. Participants will also be encouraged to contact the research team (or an identified colleague independent of the research team) with any other queries.
- Establish eligibility
  - Seven to 16 years with type 1 diabetes registered at Greater Glasgow and Clyde Paediatric diabetes service.
  - Independently ambulatory
  - Are able to undertake physical activity (i.e. have not been advised against doing more PA)
  - Do not have a severe learning disability (and therefore unable to understand the study protocol)
  - Do not have severe challenging behaviour or other needs requiring constant one to one support
- Screen participants
  Participants will we be screened for eligibility based on the inclusion/exclusion criteria.
- Check all consent has been obtained
  The RA will monitor and follow up consent forms. The chief investigator will ensure informed consent is obtained before any of the specific protocol procedures are carried out.
- Baseline measurements carried out
- Randomisation into intervention group/offer starting date (January 2016)
  Participants will be randomised into the ActivPals intervention group or the waiting control group.

Stage 4—intervention begins (February 2016–April 2016)
